# Mechanistic Studies of the Genetically Encoded Fluorescent Protein Voltage Probe ArcLight

**DOI:** 10.1371/journal.pone.0113873

**Published:** 2014-11-24

**Authors:** Zhou Han, Lei Jin, Fuyi Chen, Joseph J. Loturco, Lawrence B. Cohen, Alexey Bondar, Josef Lazar, Vincent A. Pieribone

**Affiliations:** 1 The John B. Pierce Laboratory, Inc., New Haven, Connecticut, United States of America; 2 Department of Cellular and Molecular Physiology, Yale University School of Medicine, New Haven, Connecticut, United States of America; 3 Department of Physiology and Neurobiology, University of Connecticut, Storrs, Connecticut, United States of America; 4 Center for Functional Connectomics, Korea Institute of Science and Technology, Seoul, Republic of Korea; 5 Institute of Nanobiology and Structural Biology GCRC, Academy of Sciences of the Czech Republic, Zamek 136, Nove Hrady, Czech Republic; 6 Department of Molecular Biology, University of South Bohemia, Branisovska 31, Ceske Budejovice, Czech Republic; 7 Department of Neurobiology, Yale University School of Medicine, New Haven, Connecticut, United States of America; Indiana University School of Medicine, United States of America

## Abstract

ArcLight, a genetically encoded fluorescent protein voltage probe with a large ΔF/ΔV, is a fusion between the voltage sensing domain of the *Ciona instestinalis* voltage sensitive phosphatase and super ecliptic pHluorin carrying a single mutation (A227D in the fluorescent protein). Without this mutation the probe produces only a very small change in fluorescence in response to voltage deflections (∼1%). The large signal afforded by this mutation allows optical detection of action potentials and sub-threshold electrical events in single-trials *in vitro* and *in vivo*. However, it is unclear how this single mutation produces a probe with such a large modulation of its fluorescence output with changes in membrane potential. In this study, we identified which residues in super ecliptic pHluorin (vs eGFP) are critical for the ArcLight response, as a similarly constructed probe based on eGFP also exhibits large response amplitude if it carries these critical residues. We found that D147 is responsible for determining the pH sensitivity of the fluorescent protein used in these probes but by itself does not result in a voltage probe with a large signal. We also provide evidence that the voltage dependent signal of ArcLight is not simply sensing environmental pH changes. A two-photon polarization microscopy study showed that ArcLight's response to changes in membrane potential includes a reorientation of the super ecliptic pHluorin. We also explored different changes including modification of linker length, deletion of non-essential amino acids in the super ecliptic pHluorin, adding a farnesylation site, using tandem fluorescent proteins and other pH sensitive fluorescent proteins.

## Introduction

ArcLight [1] is a genetically encoded fluorescent voltage probe is based on the voltage sensing domain (VSD) of *Ciona instestinalis* voltage sensitive phosphatase (Ci-VSP) [2] fused to the fluorescent protein (FP) super ecliptic pHluorin [3] carrying a single point mutation (A227D in the FP). ArcLight responds to voltage changes across the cell membrane of cultured HEK293 cells with large amplitude decreases in fluorescence (−30 to −40% ΔF/F, in response to a 100 mV depolarization; 1). ArcLight allows reliable detection of single action potentials and sub-threshold electric events in cultured hippocampal neurons in single trials [1] and *in vivo* in *Drosophila* [4]. It is unclear how this mutation, A227D, produced a probe which has such a large modulation of its fluorescence output in response to changes of membrane potential however it does appear to be transferable to other voltage sensors [5]. We have tested a large number of FPs in conjugation with the Ciona voltage sensitive domain but have not found one with as large modulation as the ArcLight fluorescent protein.

Many other FP voltage sensors are also based on Ci-VSP. These include: VSFP2 [6], Mermaid [7], [8], split fluorescent proteins [9], Butterfly [10] and ElectricPK [11]. The *Ciona*-based FP voltage sensors often show good membrane expression in mammalian cells [1], [6], [7], [8]. Voltage sensitive phosphatase (VSP) from other species were also exploited [5]. ArcLight variants based on chicken VSP showed fast kinetics but decreased response amplitude [5]. ASAP1 is based on chicken VSP and circularly permuted GFP [12]. It also features a fast response. Microbial rhodopsin based voltage sensors [13], [14], [15] have the most rapid kinetics so far, but the low brightness of these probes limits their use in broader applications. A new approach to combine the rapid kinetics of a fungal rhodopsin with the brightness of genetically engineered protein fluorophores resulted in a probe with fast kinetics and brightness [15], [16].

## Results

### 1. Critical residues required in the super ecliptic pHluorin in ArcLight for producing large signals

**Figure 1:**
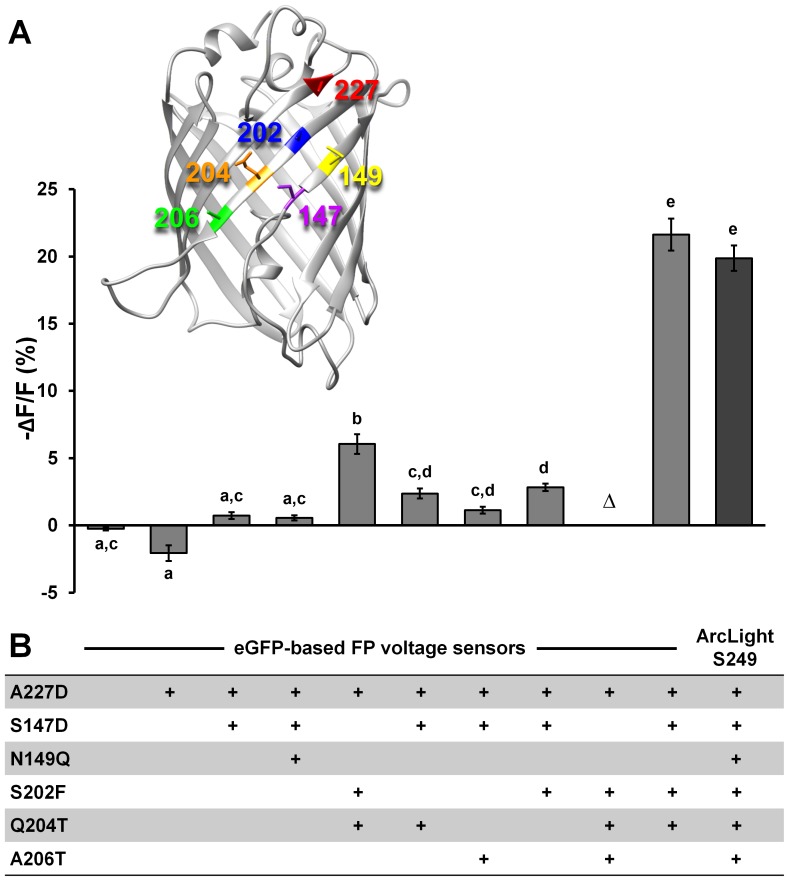
Converting an eGFP based Ciona FP voltage sensor to one like ArcLight. **A**) Comparison of the mean response amplitudes of Ciona voltage sensor domain-eGFP-S249 carrying different combinations of amino acids that are the same as those in super ecliptic pHluorin A227D. A probe with poor plasma membrane localization is indicated with the Greek letter Δ. Means with the same letter do not differ significantly (Tukey-Kramer HSD test). eGFP (n = 10), eGFP-A227D (n = 5), eGFP-A227D-S147D (n = 4), eGFP-A227D-S147D-N149Q (n = 4), eGFP-A227D-S202F-Q204T (n = 5), eGFP-A227D-S147D-S202F-Q204T (n = 4), ArcLight-S249 (n = 10). All the measurements were made in HEK293 cells (See Methods). Insert is the 3D structure of eGFP (PDB file 1EMG). The non-identical amino acids of eGFP and super ecliptic pHluorin A227D that lie on the facing surface of eGFP are colored and numbered. **B**) Mutations to eGFP and their combinations in making those probes tested in panel A are given in corresponding columns in the table. Values are means ± SEM.

Replacing of the super ecliptic pHluorin A227D in ArcLight with eGFP resulted in a probe with little voltage sensitivity [1]. eGFP differs from super ecliptic pHluorin at only nine residues (R80Q, D147S, Q149N, A163V, G175S, F202S, T204Q, T206A, H231L). We identified three residues, D147, F202 and T204, in super ecliptic pHluorin A227D, which are required for producing ArcLight signals [1]. Here, we reverse the process and introduced four critical residues to an eGFP based probe, in hopes of converting its response properties to those of ArcLight (Figure 1). We found that the new probe produced a signal with voltage sensitivity as large as that of ArcLight. eGFP based probes carrying less than a complete set of four mutations produced signals with greatly reduced amplitude (Figure 1). Neither the N149Q nor the A206T mutations are needed for generating a large signal, a result consistent with our previous observations [1].

**Figure 2:**
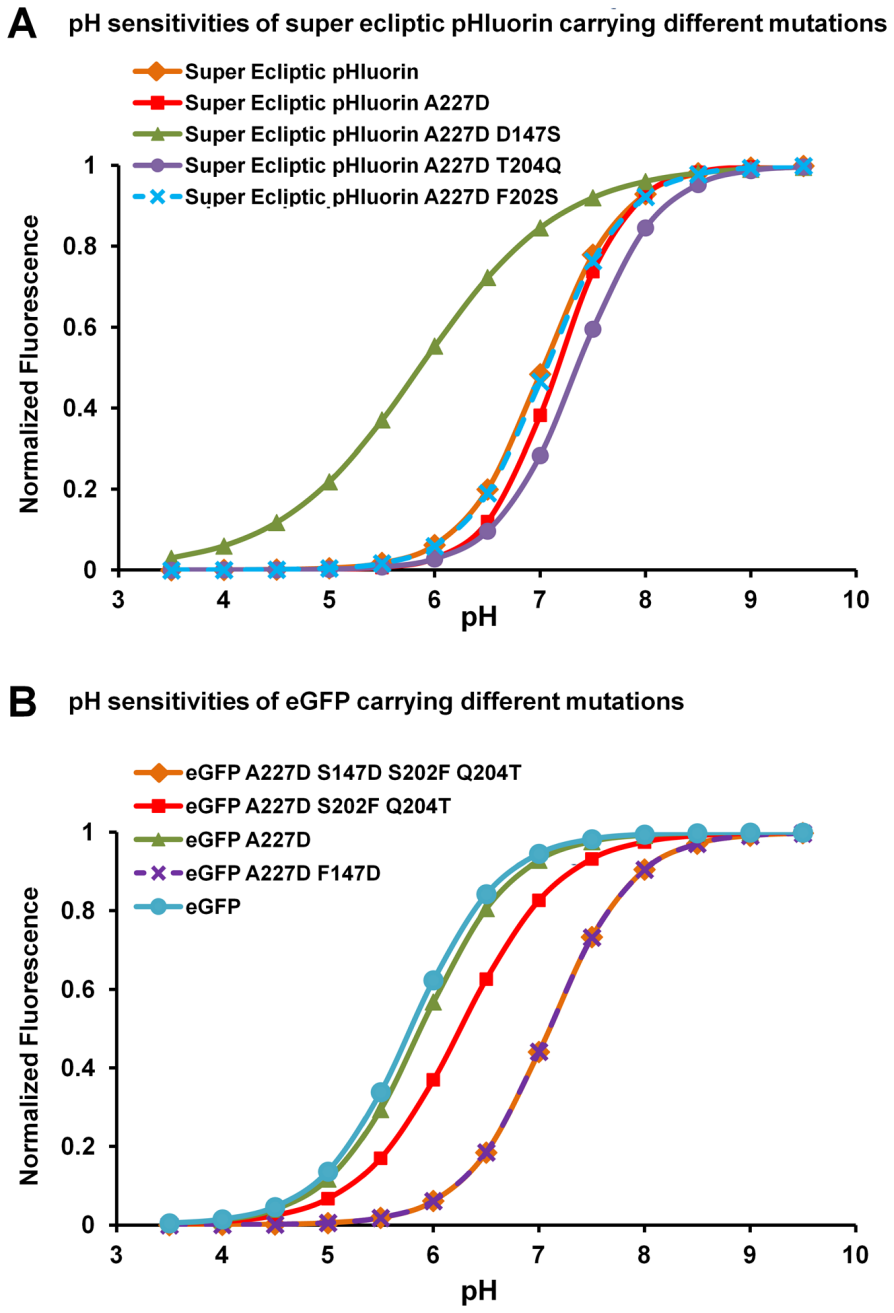
pH sensitivities of free fluorescent proteins carrying different combinations of amino acids. **A**) The fitted pH sensitivity of super ecliptic pHluorin with the indicated mutations that make it more like eGFP. **B**) The fitted pH sensitivity of eGFP with the indicated amino acids that make the eGFP more like super ecliptic pHluorin A227D.

The A227D mutation does not alter the spectral properties or pH sensitivity of the super ecliptic pHluorin [1]. We sought to determine the effect of the other 3 residues on the spectral properties or pH sensitivity of the fluorescent protein. Single mutations or a combination of mutations were introduced to the free fluorescent proteins and the emission spectra were measured. We determined the pH sensitivities of these free fluorescent proteins and found that residue D147 is responsible for the right shifted pH sensitivity of pHluorin derived fluorescent proteins. D147 alone is sufficient to change the pKa of eGFP (∼6.0) to that of super ecliptic pHluorin (∼7.1) (Figure 2A and 2B), but it is not sufficient to generate a large voltage sensitive signal (Figure 1). F202 and T204 do not significantly change the pH sensitivities of the fluorescent proteins (Figure 2A and 2B), but are needed for the large signal (Figure 1).

### 2. Is the voltage sensitivity of ArcLight caused by direct sensing of the environmental pH?

The local pH near the plasma membrane could change during the action potential or sub-threshold electric activity, due to movement of protons attracted or repelled by the electric field, or as a result of the ion fluxes that occur during these events. We had already determined [1] that pHluorin alone (without the A227D mutation) does not produce probes with a large ΔF and yet has the same pH sensitive response curve as the ArcLight FP. We sought to determine if the voltage-dependent fluorescent response of ArcLight is mediated by environmental pH changes or by the conformation changes of the *Ciona*-based voltage sensor.

**Figure 3:**
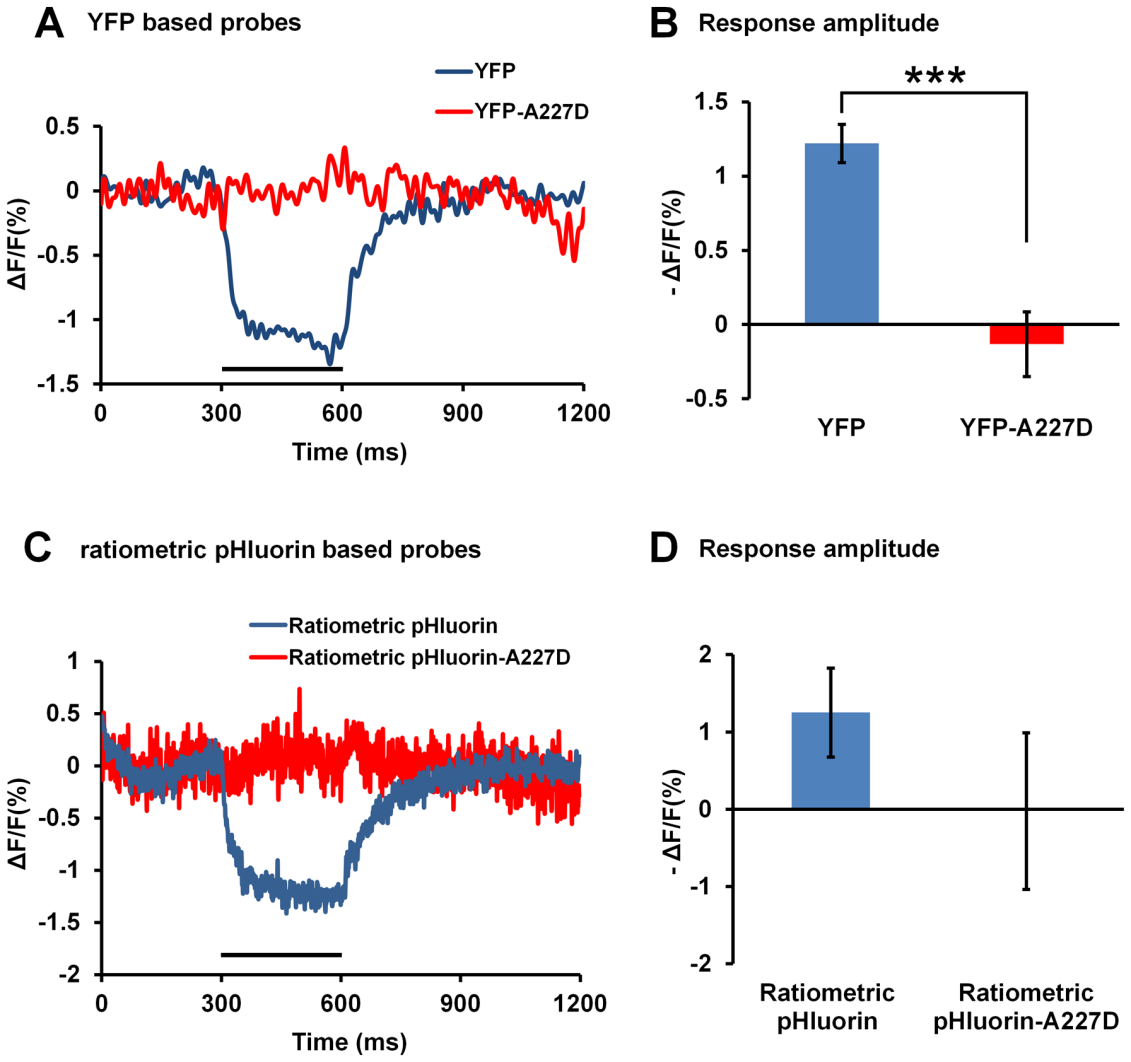
Replacing the super ecliptic pHluorin A227D in ArcLight with the pH sensitive fluorescent proteins YFP or ratiometric pHluorin. **A**) Averaged optical traces of two probes, YFP (n = 6) or YFP-A227D (n = 5) inserted at S249 of the Ciona voltage sensitive domain, in response to 100 mV depolarizations of 300 ms from the holding potential of −70 mV. The traces are processed by low pass FFT filtering of 50 Hz. **B**) The mean response amplitude of the two probes in panel A. (*** P<0.001) **C**) Averaged optical traces of two probes, ratiometric pHluorin (n = 6) or ratiometric pHluorin-A227D (n = 3) inserted at S249 of Ciona voltage sensitive domain, in response to 100 mV depolarizations of 300 ms from the holding potential of −70 mV. **D**) The mean response amplitude of the two probes shown in panel C. No significant difference was detected between the two groups. Values are means ± SEM.

First we determined if *Ciona*-based FP voltage sensors carrying other FPs with base-shifted pH sensitivity curves could result in similar signals as seen with ArcLight. We replaced the fluorescent protein in ArcLight-S249 with either YFP or ratiometric pHluorin [3]. Both fluorescent proteins have pH/fluorescence profiles that are similar to pHluorin [17]. The resulting probes exhibit less than 1.5% changes in response amplitude to a 100 mV depolarization (Figure 3A–3D). Introducing the A227D mutation reduced the signal amplitude even further (Figure 3A–3D). Thus not every pH sensitive fluorescent protein can replace super ecliptic pHluorin A227D in generating a large voltage response

**Figure 4:**
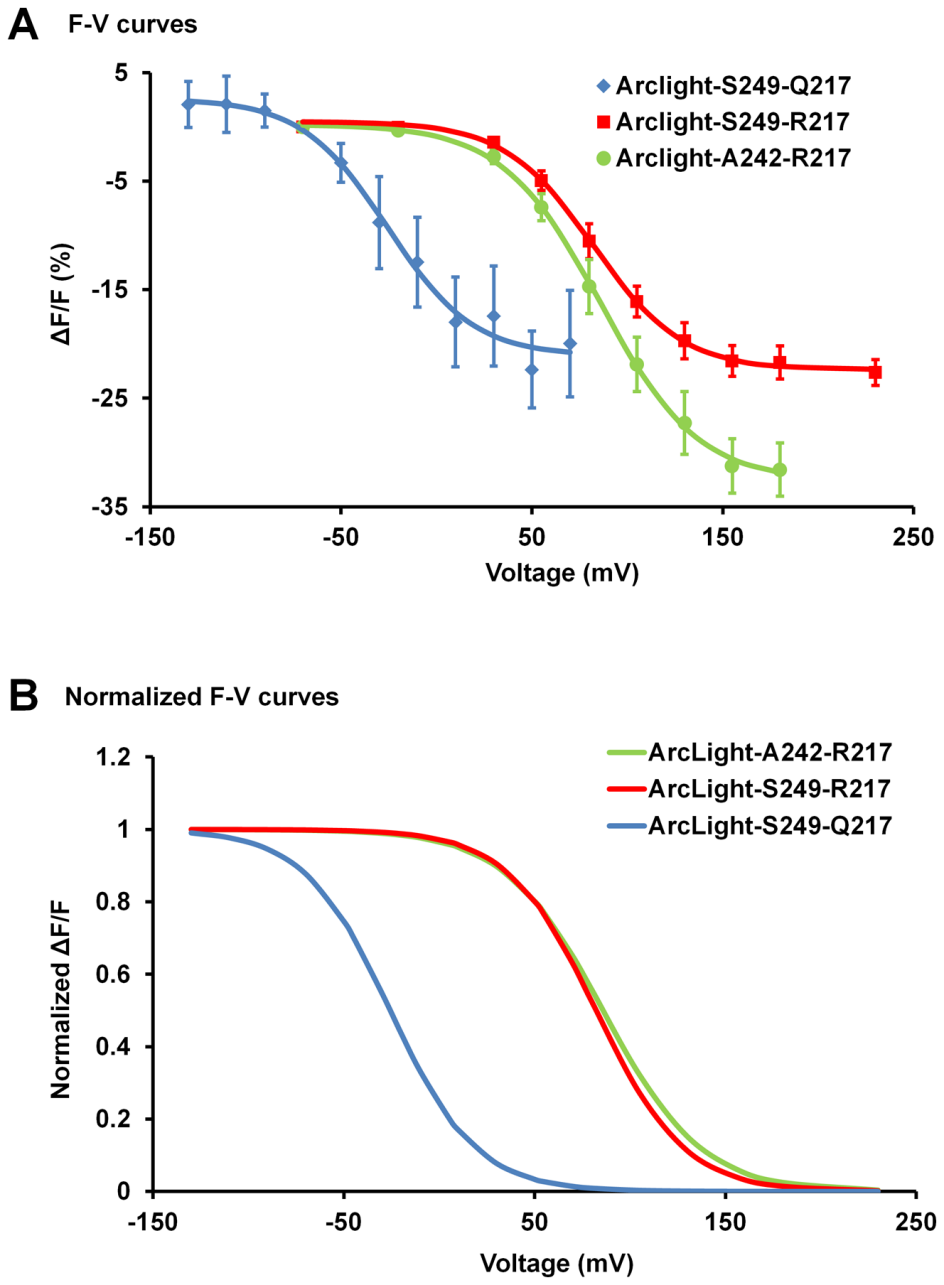
ArcLight does not simply respond to environmental pH changes. The fluorescence signals change as expected when the composition of S4 is altered. **A**) Plots of ΔF/F vs membrane voltage of three probes: ArcLight-S249-Q217, ArcLight-S249-R217 and ArcLight-A242-R217. The smooth curves are sigmoid fits. **B**) The normalized sigmoid curves of the three probes. Values are means ± SEM.

Next, we compared the F-V curves of three derivatives of ArcLight, ArcLight-249, ArcLight-249 (R217) and ArcLight-A242 (R217), whose voltage sensors are different in their voltage sensitivity. The voltage sensor of ArcLight contains a point mutation, R217Q, which changes its voltage sensitivity to a more physiologically relevant range, with a V_1/2_ at around −26 mV [18]. In comparison, the ArcLight-249 and -242 without the R217Q mutation have their V_1/2_ at around +82 mV and +86 mV, respectively. We found that these FP voltage sensors are not simply sensitive to a change in pH that results from activity because they have very different voltage sensitivities that correspond to the sensitivities of their own voltage sensors (Figure 4A and 4B) depending on differences in the S4 transmembrane helix.

### 3. Two-photon polarization microscopy study of the FP moiety movement in ArcLight

**Figure 5:**
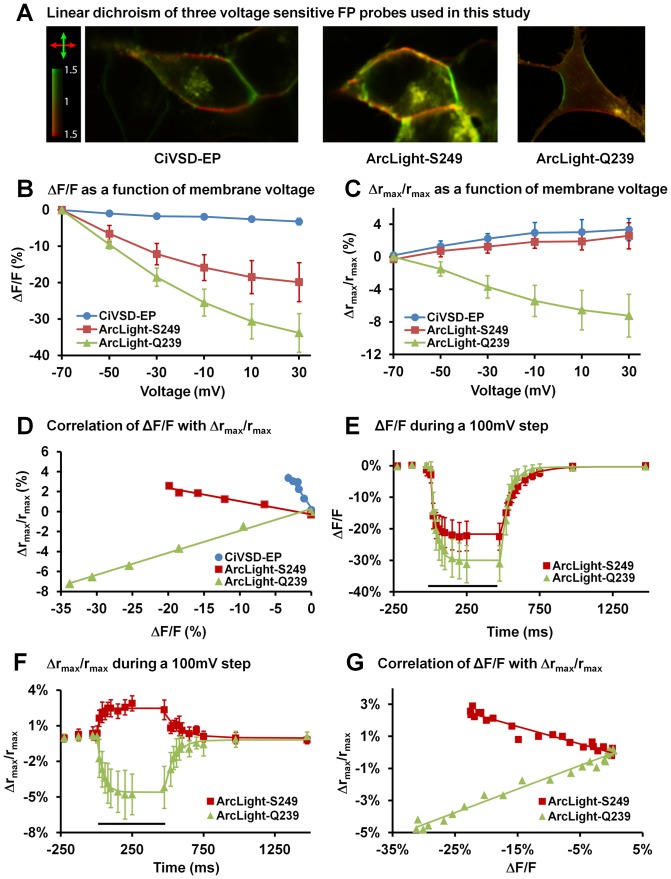
Using two-photon polarization microscopy to study the orientation and movement of the FP moiety in ArcLight. **A**) Linear dichroism of CiVSD-EP, ArcLight-S249 and ArcLight-Q239. Excess of fluorescence elicited by light polarized horizontally and vertically is shown by red and green color, corresponding to a dichroic ratio indicated by the color scale bar. **B**) ΔF/F as a function of membrane voltage. **C**) Changes in dichroic ratio (Δr_max_/r_max_) as a function of membrane voltage. **D**) Correlation of ΔF/F with Δr_max_/r_max_. **E**) Dynamics of fluorescence change during a 100 mV depolarization and repolariztion observed with two-photon polarization microscopy. **F**) Dynamics of changes in dichroic ratio (Δr_max_/r_max_) during a 100 mV depolarization and repolariztion observed with two-photon polarization microscopy. **G**) Correlation of ΔF/F with Δr_max_/r_max_ measured during the depolarization and repolarization of a 100 mV step. Values are means ± SEM.

We used a two-photon polarization microscope [19] to study the orientation and movement of the FP moiety in three similarly constructed probes: *Ciona* voltage sensitive domain-ecliptic pHluorin (CiVSD-EP), Arclight-S249 and Arclight-Q239. All three probes show similar linear dichroism (differences in absorption of light of distinct linear polarizations), indicative of the fluorophore's long axis being close to parallel to the cell membrane (Figure 5A). The two-photon F-V curves of these probes (Figure 5B) are similar to those recorded with single-photon microscopy [1]. Arclight-Q239 (−34%) and Arclight-S249 (−20%) showed much larger signal amplitudes than CiVSD-EP (−3%) in response to a 100 mV depolarization. However, changes in the dichroic ratio (Δr_max_/r_max_) of the three probes did not correlate with their fluorescence intensity changes in either signal size or sign. The Δr_max_/r_max_ of CiVSD-EP and Arclight-S249 increased by 3.4% for CiVSD-EP and 2.6% for Arclight-S249 for a 100 mV depolarization, while the Δr_max_/r_max_ of ArcLight-Q239 decreased by −7.2% (Figure 5C). The ΔF/F *vs* Δr_max_/r_max_ was linear for each probe (Figure 5D).

We also measured the dynamics of the fluorescence (Figure 5E) and dichroic ratio changes (Figure 5F) with two-photon polarization microscopy. The ΔF/F changes during a 100 mV depolarization and repolarization were fit with single exponential equations for both ArcLight-S249 (τ_on_ = 20 ms, τ_off_ = 110 ms) and ArcLight-Q239 (τ_on_ = 38 ms, τ_off_ = 70 ms). The speed of dichroic ratio change was similar (ArcLight-S249: τ_on_ = 15 ms, τ_off_ = 101 ms; ArcLight-Q239: τ_on_ = 34 ms, τ_off_ = 58 ms.). We plotted Δr_max_/r_max_ against the ΔF/F of the different time points during the depolarization and repolarization, again, the Δr_max_/r_max_ *vs* ΔF/F appears linear during the voltage transitions (Figure 5G).

### 4. Modification to the linker length of ArcLight

**Figure 6:**
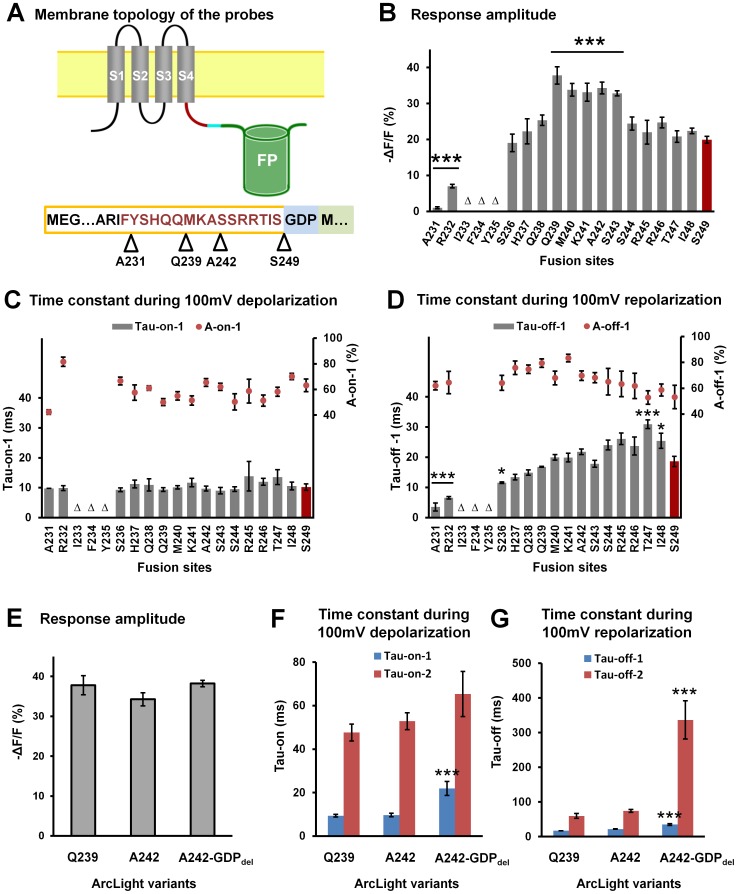
The relationship of ArcLight response amplitude to linker length. **A**) Top: Schematic topology of the fusion protein in the plasma membrane; Bottom: The linker length modified derivatives of ArcLight. The sequence of the Ciona voltage sensitive domain and linker are framed in orange, the linker sequence is indicated with red; the three amino acids sequence “GDP” was introduced during plasmid construction and is shaded with blue; the sequence of the super ecliptic pHluorin A227D is shaded with green. The range of linker modification is from A231 to S249. **B**) The mean response amplitude of the linker length modified derivatives of ArcLight. Probes with poor plasma membrane localization are indicated with the Greek letter Δ. Groups that have significant difference with ArcLight S249 were indicated with the asterisks (*** P<0.001). n = A231: 6; R232: 7; S236: 8; H237: 5; Q238: 6; Q239: 7; M240: 8; K241: 7; A242: 7; S243: 7; S244: 8; R245: 5; R246: 7; T247: 8; I248: 8; S249: 10. **C**) The mean tau-on-fast component (Tao-on-1)and its amplitude contribution (A-on-1) in the double exponential fitting of the fluorescence response during depolarization. No significant difference was detected between these groups. n = A231: 1; R232: 7; S236: 7; H237: 5; Q238: 6; Q239: 6; M240: 8; K241: 7; A242: 6; S243: 7; S244: 8; R245: 4; R246: 7; T247: 8; I248: 8; S249: 9. **D**) The tau-off-fast component (Tau-off-1) and its amplitude contribution (A-off-1) during repolarization. Groups that have significant difference with ArcLight S249 were indicated with the asterisks (* P<0.05; *** P<0.001). n = A231: 4; R232: 6; S236: 7; H237: 4; Q238: 5; Q239: 6; M240: 8; K241: 7; A242: 6; S243: 7; S244: 5; R245: 4; R246: 6; T247: 8; I248: 7; S249: 10. **E**) The mean response amplitude of ArcLight-Q239 (n = 7), ArcLight-A242 (n = 7) and ArcLight-A242-GDP_del_ (n = 4). No significant difference was detected between these groups. **F**) The mean fast (Tau-on-1) and slow components (Tau-on-2) for the double exponential fitting of the fluorescence response during depolarization. ArcLight-Q239 (n = 6), ArcLight-A242 (n = 6) and ArcLight-A242- GDP_del_ (n = 4). Groups that have significant difference with ArcLight A242 were indicated with the asterisks (*** P<0.001). **G**) The mean fast (Tau-off-1) and slow components (Tau-off-2) for the double exponential fitting of the fluorescence response during repolarization. ArcLight-Q239 (n = 6), ArcLight-A242 (n = 6) and ArcLight-A242- GDP_del_ (n = 4). Groups that have significant difference with ArcLight A242 were indicated with the asterisks (*** P<0.001). Values are means ± SEM.

Nineteen linker length derivatives of ArcLight were generated by inserting the super ecliptic pHluorin A227D after each residue between A231 and S249 of the Ciona voltage sensitive phosphatase sequence (Figure 6A). Three of these derivatives, I233, F234 and Y235, did not express on the plasma membrane in HEK293 cells. The five ArcLight derivatives previously reported, i.e. Q239, M240, K241, A242 and S243 [1], exhibited the largest voltage sensitivity, while probes with greater or shorter linker lengths display a gradual reduction in voltage response (Figure 6B). The dynamics of all these probes are best fit with double exponential equations during depolarization and repolarization. None of the new linker length modified derivatives had “on” response kinetics significantly different than the previously reported five ArcLight derivatives (Figure 6C and Table 1). However, the time constants (tau) of the fast component during repolarization decreased with shorter linker lengths (Figure 6D).

**Table 1:** Dynamic properties of the linker modified ArcLight derivatives.

Insertion sites	Tau-on-1 (ms)	A-on-1 (%)	Tau-on-2 (ms)	Tau-off-1 (ms)	A-off-1 (%)	Tau-off-2 (ms)
A231	10	42	55	4±1	62±3	35±19
R232	10±1	82±4	121±28	7±1	64±9	19±2
S236	9±1	67±3	70±5	12±1	64±6	60±9
H237	11±1	58±6	69±9	13±1	76±5	82±6
Q238	11±2	61±2	79±30	15±1	75±3	87±8
Q239	9±1	50±3	48±4	17±1	79±3	60±7
M240	10±1	55±3	52±4	20±1	68±5	77±11
K241	12±1	52±4	51±6	20±1	83±3	68±10
A242	10±1	65±3	53±4	22±1	70±4	74±4
A243	9±1	62±3	44±5	18±1	68±4	53±9
S244	10±1	50±6	64±8	24±2	65±10	125±17
R245	14±5	59±9	68±9	26±2	63±10	104±5
R246	12±1	51±4	60±5	24±3	62±10	80±11
T247	14±3	58±4	106±20	31±2	53±5	137±12
I248	11±1	70±2	46±5	25±3	59±5	116±37
S249	10±1	63±5	47±4	19±2	53±9	62±9

The dynamics of all tested probes of linker modified ArcLight derivatives during depolarization and repolarization were fit with double exponential equations. The time constant (tau) of the fast and slow components and their amplitude contribution (A) are provided in the table. Values are means ± SEM.

The linker sequences of all ArcLight derivatives previously reported include three additional amino acids, glycine, aspartic acid and proline (GDP), translated from a BamHI restriction site introduced during plasmid construction (Figure 6A). We created a new construct, ArcLight-A242-GDP_del_, which lacks the three extra amino acids (GDP) and compared its voltage response and dynamics to probes with similar linker lengths. ArcLight-A242-GDP_del_ showed similar voltage sensitivity as ArcLight-A242 and ArcLight-Q239 (Figure 6E). However, its response dynamics were slower during both depolarization and repolarization (Figure 6F and 6G).

### 5. Deletion of non-essential residues at the N- or C-terminus of the fluorescent protein in ArcLight

**Figure 7:**
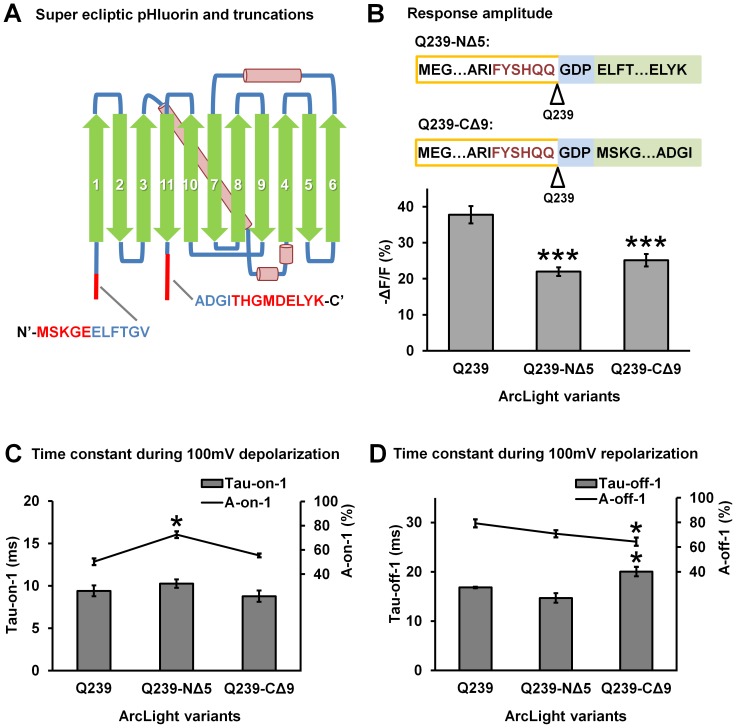
The effect of deletion of non-essential residues at the N- or C-terminus of super ecliptic pHluorin A227D on the response properties of ArcLight. **A**) The schematic structure of super ecliptic pHluorin A227D and truncations. The N-terminal segment deleted in ArcLight-Q239-NΔ5 and the C-terminal segment deleted in ArcLight-Q239-CΔ9 are indicated with red. **B**) Top: sequence of ArcLight-Q239-NΔ5 and ArcLight-Q239-CΔ9. Bottom: The mean response amplitude of ArcLight-Q239 (n = 7), ArcLight-Q239-NΔ5 (n = 6) and ArcLight-Q239-CΔ9 (n = 8). Groups that have significant difference with ArcLight Q239 were indicated with the asterisks (*** P<0.001). **C**) The mean fast component (Tau-on-1) and its amplitude contribution (A-on-1) in the double exponential fitting of the fluorescence response during depolarization. ArcLight-Q239 (n = 6), ArcLight-Q239- NΔ5 (n = 6) and ArcLight-Q239- CΔ9 (n = 8). Groups that have significant difference with ArcLight Q239 were indicated with the asterisks (* P<0.05). **D**) The mean fast component (Tau-off-1) and its amplitude contribution (A-off-1) in the double exponential fitting of the fluorescence response during repolarization. ArcLight-Q239 (n = 6), ArcLight-Q239- NΔ5 (n = 6) and ArcLight-Q239- CΔ9 (n = 8). Groups that have significant difference with ArcLight Q239 were indicated with the asterisks (* P<0.05). Values are means ± SEM.

The N- and C- termini of eGFP are not involved in formation of the β-barrel structure and are relatively flexible. It was shown that up to five amino acids at the N-terminus and nine amino acids at the C-terminus can be deleted from the sequence of eGFP without affecting its fluorescent properties [20]. Super ecliptic pHluorin is homologous to eGFP. We determined if deletion of these non-essential amino acids in the super ecliptic pHluorin A227D of ArcLight affects the response properties of the probe. We made derivatives of ArcLight-Q239 with deletion of either the five amino acids from the N-terminus (Q239-NΔ5) or the nine amino acids from the C-terminus (Q239-CΔ9) of the super ecliptic pHluorin A227D (Figure 7A). The two new probes had smaller optical responses (∼20% compared with ∼40% ΔF/F for ArcLight-Q239) (Figure 7B). The time constants of the depolarization and repolarization response of these two derivatives were not significantly faster, with tau-on-1 around 10 ms and tau-off-1 around 15–20 ms (Figure 7C and 7D).

### 6. Farnesylation of the fluorescent protein in ArcLight

**Figure 8:**
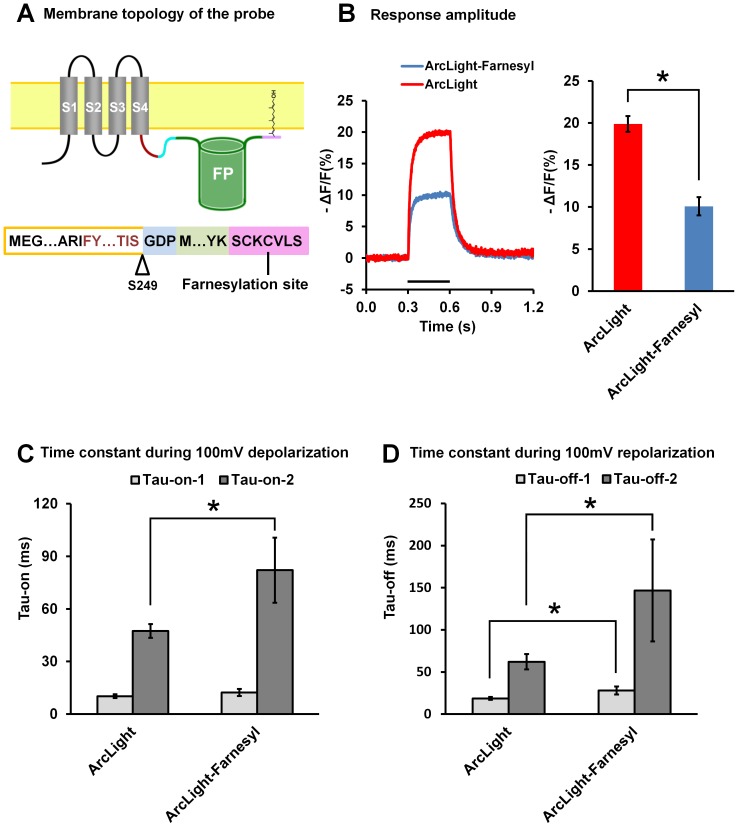
The effect of farnesylation at the C-terminus of super ecliptic pHluorin A227D on the response properties of ArcLight. **A**) Top: Schematic topology of ArcLight-S249 with a farnesylation site. Bottom: Sequence of ArcLight-S249 with a farnesylation site. The farnesylation site added to the C-terminus of the fluorescent protein is shaded with pink. **B**) Left: Non- filtered, averaged optical responses of ArcLight-S249 (red, n = 10 cells; single trial for each cell) and ArcLight-S249 with a farnesylation site (blue, n = 6 cells; single trial for each cell) to +100 mV depolarizations of 300 ms from a holding potential of −70 mV. In this and subsequent figures, the timing of the depolarization is indicated with the black line. Right: The mean response amplitude of ArcLight-S249 (n = 10) and ArcLight-S249 with a farnesylation site (n = 6). (* P<0.05) **C**) The mean fast (Tau-on-1) and slow components (Tau-on-2) of the “on” dynamics of ArcLight-S249 (n = 9) and ArcLight-S249 with a farnesylation site (n = 6) during depolarization fitted with a double exponential equation. (* P<0.05) **D**) The mean fast (Tau-off-1) and slow components (Tau-off-2) of the “off” dynamics of ArcLight-S249 (n = 10) and ArcLight-S249 with a farnesylation site (n = 3) during repolarization fitted with a double exponential equation. (* P<0.05). Values are means ± SEM.

The original ArcLight has the super ecliptic pHluorin anchored to the plasma membrane only by the linker to the Ciona voltage sensor domain. We determined the effect of adding a farnesylation site to the C-terminal of the probe (Figure 8A). The farnesyl group anchors the fluorescent protein to an additional site of the plasma membrane and therefore may change the response properties of the probe. We found that the sensitivity of the probe with the farnesylation site is reduced by half (Figure 8B), and the taus of double exponential fitting are also larger than those of ArcLight (Figure 8C and 8D).

### 7. Using tandem fluorescent proteins in ArcLight

**Figure 9:**
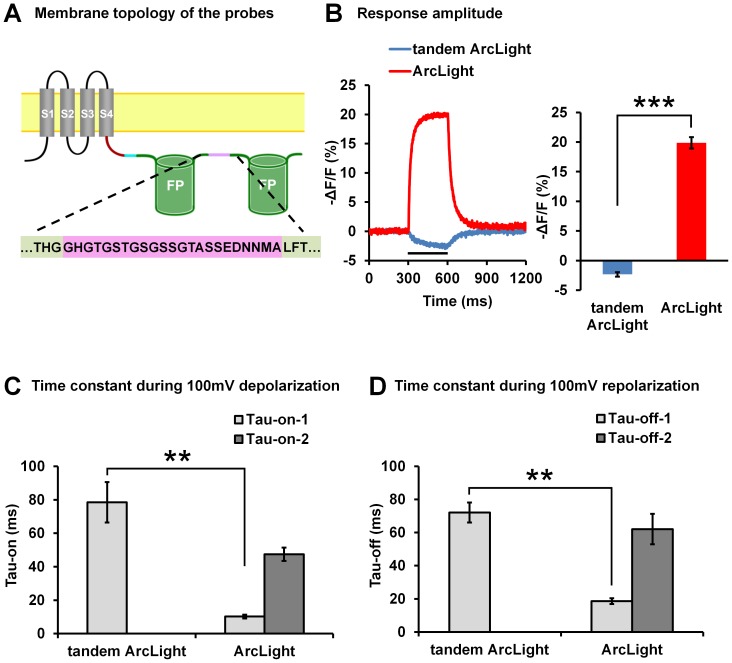
The effect of using tandem super ecliptic pHluorin A227D on the response properties of ArcLight. **A**) Schematic topology of ArcLight-S249 with tandem fluorescent proteins (tandem ArcLight-S249). The linker sequence between the two fluorescent proteins is shaded with pink. Non-essential amino acids “MDELYK” at the C-terminus of the front fluorescent protein and “MSKGEE” at the N-terminus of the back fluorescent protein were deleted in the probe. **B**) Left: Non-filtered, averaged optical responses of ArcLight-S249 (red, n = 10 cells; single trial for each cell) and tandem ArcLight-S249 (blue, n = 4 cells; single trial for each cell) to 100 mV depolarizations of 300 ms from the holding potential of −70 mV. Right: The mean response amplitude of ArcLight-S249 (n = 10) and tandem ArcLight-S249 (n = 4). (*** P<0.001) **C**) The dynamics of tandem ArcLight-S249 (n = 4) during depolarization is best fit with a single exponential equation, the mean tau of tandem ArcLight-S249 is compared with the fast (Tau-on-1) and slow components (Tau-on-2) of ArcLight-S249 (n = 9). (** P<0.005) **D**) The mean tau of tandem ArcLight-S249 (n = 4) during repolarization compared with the fast (Tau-off-1) and slow components (Tau-off-2) of ArcLight-S249 (n = 10). (*** P<0.005). Values are means ± SEM.

One possible mechanism by which ArcLight produces a large response is intermolecular dimerization between FPs in two adjacent ArcLight molecules. Introduction of a tandem FP in a single molecule may result in intramolecular dimerization [21] at the expense of intermolecular dimerization. A new probe, tandem ArcLight, was made with a tandem repeat of super ecliptic pHluorin A227D (Figure 9A). The linker length and composition were copied from those that are used in tdTomato [22]. The voltage dependent fluorescence response of the tandem ArcLight probe was inverted from a depolarization dependent decrease in fluorescence to an increase and the signal size was greatly reduced to +2% (Figure 9B). The dynamics of the tandem ArcLight are better fit with single exponential. The time constants (taus) of tandem ArcLight were slower than the slow components of ArcLight (Figure 9C and 9D).

## Methods

### Molecular biology

Mutations and modification to the original ArcLight probe were introduced by using the QuickChange II XL site-directed mutagenesis kit (Agilent Technologies, INC., CA). All DNA constructs were verified by sequencing using the dye-termination method (W. M. Keck Foundation, Biotechnology Resource Laboratory, Yale University, CT)

### Fluorescent protein purification

Expression constructs were generated by inserting PCR amplified fragments of fluorescent proteins' cDNA into the pCR4Blunt TOPO vector (Invitrogen, NY). This procedure introduces a 6×His tag to the N-terminus of the fusion proteins to allow affinity purification. Top10 bacteria (Invitrogen, NY) were transformed with the expression constructs and fusion proteins were purified with His-Select Nickel Affinity Gel (Sigma-Aldrich, MO), following the manufacturer's instructions. The purified proteins were concentrated with Amicon Ultra-15 centrifugal filters (MWCO 10,000, Millipore, MA), dialyzed against 100 mM sodium phosphate buffer, pH 7.4 and stored at 4°C.

### Spectrofluorimetry

Fluorescence emission spectra of the fusion proteins were measured with a Horiba Jobin Yvon Fluorolog 3 spectrophotometer (Horiba, Japan). In order to determine pH-dependent fluorescence, purified proteins were diluted to a concentration of 0.36 µM in pH adjusted buffers containing 100 mM NaCl, 1 mM CaCl_2_ and 1 mM MgCl_2_. The pH of the buffers was adjusted with MES (for pH 3.5, 4.5 and 5.5), HEPES (for pH 6.5 and 7.5) or Bicine (for pH 8.5 and 9.5) to a final concentration of 25 mM of the buffers. To determine the protein concentration, the protein was denatured in 0.1N of NaOH and absorption at 280 nm was measured using a Beckman Coulter DU 730 uv/vis spectrophotometer (Beckman Coulter, Inc. CA).

### Cell culture

HEK293 cells (ATCC, VA) were maintained in Dulbecco's Modified Eagle Medium (High Glucose; DMEM; Invitrogen, NY) supplemented with 8% fetal bovine serum (FBS; Invitrogen, NY). Cortical neurons were isolated from E18 rat embryos and maintained in Neurobasal medium with 0.5 mM Glutamax-I and 1 ml of B-27 supplement (Invitrogen, NY) per 50 ml of cultured medium. Cells were plated on coverslips coated with poly-D-lysine hydrobromide (MP Biomedicals, OH) and kept in an incubator at 37°C with 5% CO_2_. Transient transfection was accomplished by using half of the manufacturer's recommended amount of DNA (2 µg per 35 mm dish or 0.4 µg per 12 mm coverslip in 24-well dish) and Lipofectamine2000 (1 µl per 12 mm coverslip; Invitrogen, NY).

### Electrophysiology

Microelectrode recordings were performed in a perfusion chamber with the bath temperature kept at 33°C by a temperature controller. The bath solution contained: 150 mM NaCl, 4 mM KCl, 2 mM CaCl_2_, 1 mM MgCl_2_, 5 mM D-glucose, and 5 mM HEPES, pH 7.4. We used a 3–5 MΩ glass patch pipettes (capillary tubing with 1.5/0.75 mm OD/ID-World Precision Instruments, FL) that were pulled on a P-97 Flaming/Brown type micropipette puller (Sutter Instrument Company, CA). The pipette solution contained 120 mM K-aspartate, 4 mM NaCl, 4 mM MgCl_2_, 1 mM CaCl_2_, 10 mM EGTA, 3 mM Na_2_ATP and 5 mM HEPES, pH 7.2. Voltage-clamp recordings in the whole-cell configuration were performed using a Patch Clamp PC-505B amplifier (Warner Instruments, CT) with a holding potential of −70 mV. The amplifier outputs were recorded using NeuroPlex (RedShirtImaging, LLC, GA).

### Wide field imaging

Whole-cell patch clamped cells were imaged with a Nikon Eclipse E6000FN upright microscope with a water immersion objective, Nikon Fluor 60×/1.00 N.A. A MLL-FN-473 nm 50 mW (Changchun New Industries Optoelectronics Tech. Co., Ltd., China) was used as the excitation light source. The laser light was coupled to the microscope by a multi-mode fiber coupler (Siskiyou, OR), a quartz light guide and an Achromatic EPI-Fluorescence Condenser (Till Photonics, NY). The filter cube contains a dichroic mirror 505DCXR and an emission filter HQ510LP (Chroma, Bellows Falls, VT). The fluorescence image was demagnified by an Optem zoom system, A45699 (Qioptiq Inc, NY) and projected onto the 80×80 pixel chip of a NeuroCCD-SM camera (RedShirtImaging, LLC) controlled by NeuroPlex software. The images were recorded at a frame rate of 1000 fps.

### Data processing

NeuroPlex software was used to view the image sequences and output optical and electrophysiological recordings. The % ΔF/F was calculated by first subtracting the dark offsets from all frames, then the average of a region of interest in each frame (F) is subtracted from the average of the region taken from one hundred frames prior to the event of interest (F0) and this value is then divided by F0, i.e. % ΔF/F = ((F−F0)/F0)*100. Bleaching in individual trials was corrected using the Exponential Subtraction function in NeuroPlex software. If needed, traces from individual cells were smoothed by a Gaussian low pass filter in NeuroPlex software, and the averaged trace from multiple cells was smoothed by the fast Fourier transform (FFT) low pass filter in Origin 9.1 (OriginLab, MA). The data were further processed and statistically analyzed with Origin 9.1 and Excel (Microsoft, WA) for the tau values. The probe dynamics were fit with either a single or double exponential equation.

The voltage sensitivity of the fluorescence change (ΔF/F vs V plot) was analyzed with the Boltzmann equation:
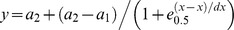
where a_1_ and a_2_ are constants, τ1 and τ2 are time constants in ms, x_0.5_ is the membrane potential in mV at half maximal ΔF/F, and dx is the slope.

The normalized ΔF/F vs V plot is calculated from the Boltzmann fit:
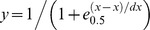



### Statistics

Measurements of each set of data are given as mean ± SEM with n equals to the number of independent experiments. For comparison of means between two independent groups, Two-tailed Student t test was performed at significant level (α) of 0.05. Before performing the Student t test, however, the Shapino-Wilk test was performed to determine if the data within each group follow the normal (Gaussian) distribution and equal variance was tested between groups. If variance between two groups is not equal, the Welch's correction was used in the t test. If the data do not follow normal distribution, the Mann-Whitney U nonparametric test was used. For comparison of means between three or more groups, one-way analysis of variance (ANOVA) was performed, followed by Post hoc Tukey-Kramer HSD multiple comparisons. A nonparametric test (Kruskal-Wallis ANOVA) was used when any group of data did not present in a normal distribution. The analyses were performed using the Origin 9.1 software (OriginLab, MA).

### Two-photon polarization microscopy (2PPM)

Two-photon polarization microscopy was performed on a laser-scanning microscope iMic (Till Photonics GmbH, Germany), equipped with a Yanus beam scanner (Till Photonics) and a tunable pulsed titanium:sapphire laser (Chameleon Ultra II with GVD compensation, Coherent, CA) operated at 960 nm. The objective was a UApoPlan/IR ×60, numerical aperture (NA) 1.2 water-immersion objective lens (Olympus Corp., Japan). We used a long-pass dichroic mirror (Q565LP, Chroma Technology Corp, VT) and an emission filter (520/35, Semrock, NY). Fluorescence was detected by a photomultiplier (R6357, Hamamatsu Photonics, Japan), operated at 700–900 V, providing 16-bit output. A CCD camera Imago QE (Till Photonics) was used for transmitted light imaging.

A rapid polarization modulator RPM-2P (Innovative Bioimaging L.L.C., TX) synchronized with scanning of the microscope was used to alternate the polarization of the excitation beam between horizontal and vertical orientation between acquisition of subsequent pixels. For combined electrophysiology/two-photon polarization microscopy experiments, electrodes were prepared from GC150T-10 borosilicate capillaries (Harvard Apparatus, MA), using a PC-10 puller (Narishige, Japan), and mounted in an MP-225 micromanipulator (Sutter Instrument, CA). Voltage pulses were delivered using the EPC10 amplifier (HEKA Elektronik, Germany) controlled by PatchMaster software (HEKA).

Raw two-photon polarization microscopy images were processed as described previously (Lazar, Bondar et al. 2011) to yield a pair of images acquired with distinct polarizations of the excitation beam. For two-photon polarization microscopy voltage imaging, fluorescence of a manually selected horizontal region (within 5° of the horizontal direction) of the plasma membrane was used for analysis. After background subtraction, average fluorescence intensities F_h_ and F_v_, acquired with the horizontal and vertical excitation polarization, respectively, were used to calculate the total fluorescence F (F = F_h_+F_v_) and the dichroic ratio r (r = F_h_/F_v_). At least 10 cells were used for each measurement.

## Discussion

We identified four residues, D147, F202, T204 and D227 within the super ecliptic pHluorin, which are critical for the large ArcLight response amplitude [1]. Here we demonstrated that a similarly constructed probe based on eGFP also exhibits large response amplitude if all four critical residues are present (Figure 1). We found that residue D147 alone is responsible for the base-shifted (relative to eGFP) pH sensitivity of super ecliptic pHluorin (Figure 2), but D147 by itself is not enough to result in a large ΔF/ΔV signal when introduced to an eGFP based probe (Figure 1). These four critical residues are all located in close vicinity and are on one side of the β-barrel surface (Figure 1). This side of the surface is also the dimerization contact surface in avGFP [21]. Further investigation is needed to determine how these mutations coordinate. Intriguingly, the ArcLight probe carrying tandem fluorescent proteins had a greatly reduced response amplitude and a change in response direction (Figure 9). It is possible that the tandem super ecliptic pHluorins in this ArcLight variant form an intramolecular dimer and that this dimerization may inhibit the fluorescence changes of the probe by interfering with intermolecular dimerization or association with the plasma membrane.

Similarly constructed probes carrying pH sensitive fluorescent proteins other than super ecliptic pHluorin A227D, i.e. the YFP and ratiometric pHluorin, did not have large voltage dependent changes in fluorescence (Figure 3). These new probes produced only small response amplitude. Introduction of the A227D mutation only served to further decrease their signal sizes. These results (together with above described result of an eGFP based probe carrying the S147D mutation) show that pH sensitivity of the fluorescent protein alone is not sufficient to produce a large signal. Furthermore, the *Ciona* protein carrying super ecliptic pHluorin does not exhibit a large response amplitude without introducing the A227D mutation [1]. We also demonstrated that shifting the voltage sensitivity of the Ciona voltage sensor by introducing mutations to the S4 domain also shifts the fluorescence response of ArcLight (Figure 4). The correlation between the voltage sensitivity and the fluorescence changes provides a third line of evidence that ArcLight does not simply sense a pH changes that results from the membrane potential change.

We used two-photon polarization microscopy, an anisotropy method, to examine changes in the FPs orientation with changes in voltage and altered amino acids at the linker domain. Two-photon polarization microscopy allows sensitive observations of changes in orientation of fluorescent moieties with respect to the cell membrane [19]. In two-photon polarization microscopy, molecular orientation can be characterized by the maximum dichroic ratio *r_max_*: the ratio of fluorescence intensities (Fh/Fv) observed using two perpendicular polarizations (horizontal and vertical in the frame of the image) of the excitation beam. For a horizontally oriented section of the cell membrane, the *r_max_* is largest (theoretically infinite) when the two-photon excitation pseudo-transition dipole moment of the fluorescent moiety is oriented parallel to the plasma membrane, and smallest (theoretically zero) when the pseudo- transition dipole moment is perpendicular to the membrane. We found that the fluorescent protein in all three tested probes undergoes voltage-induced reorientation (Figure 5). In response to cell membrane depolarization, CiVSD-EP and ArcLight-S249 showed similar, small *r_max_* increases. This indicates that these two FPs' pseudo- transition dipole moments rotated slightly towards the plasma membrane. Interestingly, although the two constructs show similar changes in *r*, ArcLight-S249 exhibits a much larger fluorescence decrease than CiVSD-EP. In contrast to ArcLight-S249 and CiVSD-EP, Arclight-Q239 showed a decrease in *r_max_*, indicating that its pseudo- transition dipole moment rotated away from the plasma membrane, and the *r_max_* change was larger than in CiVSD-EP and ArcLight-S249. Thus, although the three constructs share a decrease in fluorescence intensity upon depolarization, the underlying molecular rearrangements are distinct, implying that the observed changes in fluorescence intensity are not the result of changes in the orientation of an inert β-barrel. However, the changes in *r_max_* and in fluorescence intensity closely correlate in temporal dynamics, implying a close mechanistic connection between the FP reorientation and the change in fluorescence intensity.

The response amplitude of ArcLight increases if the fluorescent protein is inserted at positions along the linker closer to the S4 transmembrane domain [1] than in a position more C-terminal. In this study, we scanned the full linker region from A231 to S249 by inserting the super ecliptic pHluorin A227D after each of the linker amino acids. We determined that there is a “sweet spot” (Q239 to A242) in the length of this linker outside of which the response amplitude is decreased (Figure 6). This drop off is precipitous as the linker length is decreased and gradual as it is increased beyond the optimal. Three of these derivatives, I233, F234 and Y235, with the fluorescent protein inserted close to the S4 domain did not traffic well to the plasma membrane. We also found that removing the three extra amino acids, glycine, aspartic acid and proline (GDP) in the linker region of all ArcLight derivatives did not significantly affect the signal size but did slow the response of ArcLight (Figure 6).

We explored several protein modifications which may change the mobility/orientation/dimerization of the FP in ArcLight. These included removing non-essential, flexible residues at the N- or C-termini of the super ecliptic pHluorin A227D (Figure 7), adding an anchoring farnesylation site to the C-terminal end of the FP (Figure 8), and using tandem super ecliptic pHluorin A227D (Figure 9). None of these approaches has improved the probes response characteristics. Instead, these modifications tend to decrease the response amplitude and slow down the response kinetics of the probes (Figure 7, 8 and 9). It is not clear why these modification cause deterioration of the ArcLight response properties.

The robust ArcLight fluorescent response may result from the modulatory effect of mutation A227D on the β-barrel structure of the FP, to which deformation may be induced by movement of the S1–S4 domains. The loosening of the β-barrel in turn allows penetration by a H^+^ ion, leading to fluorophore quenching analogous to that observed upon a pH change, and thus to a decrease in fluorescence intensity. However, this hypothesis is not supported by the observation that the fluorescent response is greatly reduced with ArcLight variants featuring obligatory intramolecular dimerization of FPs (tandem FPs, Figure 9) or fixation of the FP position (by farnesylation, Figure 8). Alternatively or concomitantly, ArcLight FPs may undergo a voltage dependent intermolecular dimerization and de-dimerization or association/disassociation with the plasma membrane. The hyperpolarization position of the S4 may allow FPs of two ArcLight subunits to dimerize, producing a fluorescent dimer. If this were true we would expect to see a membrane concentration dependent effect. Upon depolarization and net outward movement of the S4 domain, this dimerization may be disrupted, producing reduced output monomers. In fact, many other FPs are known to show reduced fluorescence output upon monomerization [23]. Alternatively depolarization dependent movements may reversibly alter the FPs association with the plasma membrane.
